# 3D bioprinting of liver models: A systematic scoping review of methods, bioinks, and reporting quality

**DOI:** 10.1016/j.mtbio.2024.100991

**Published:** 2024-02-15

**Authors:** Ahmed S.M. Ali, Dongwei Wu, Alexandra Bannach-Brown, Diyal Dhamrait, Johanna Berg, Beatrice Tolksdorf, Dajana Lichtenstein, Corinna Dressler, Albert Braeuning, Jens Kurreck, Maren Hülsemann

**Affiliations:** a Department of Applied Biochemistry, Institute of Biotechnology, Technische Universität Berlin, Germany; b Berlin Institute of Health (BIH) @Charité, QUEST Center for Responsible Research, Berlin, Germany; c German Federal Institute for Risk Assessment (BfR), Department Food Safety, Berlin, Germany; d Charité – Universitätsmedizin Berlin, Corporate Member of Freie Universität Berlin and Humboldt Universität zu Berlin, Medical Library, Germany

**Keywords:** 3D bioprinting, Bioink, Hepatocytes, HepG2, Xeno-free, 3R

## Abstract

**Background:**

Effective communication is crucial for broad acceptance and applicability of alternative methods in 3R biomedical research and preclinical testing. 3D bioprinting is used to construct intricate biological structures towards functional liver models, specifically engineered for deployment as alternative models in drug screening, toxicological investigations, and tissue engineering. Despite a growing number of reviews in this emerging field, a comprehensive study, systematically assessing practices and reporting quality for bioprinted liver models is missing.

**Methods:**

In this systematic scoping review we systematically searched MEDLINE (Ovid), EMBASE (Ovid) and BioRxiv for studies published prior to June 2^nd^, 2022. We extracted data on methodological conduct, applied bioinks, the composition of the printed model, performed experiments and model applications. Records were screened for eligibility and data were extracted from included articles by two independent reviewers from a panel of seven domain experts specializing in bioprinting and liver biology. We used RAYYAN for the screening process and SyRF for data extraction. We used R for data analysis, and R and Graphpad PRISM for visualization.

**Results:**

Through our systematic database search we identified 1042 records, from which 63 met the eligibility criteria for inclusion in this systematic scoping review. Our findings revealed that extrusion-based printing, in conjunction with bioinks composed of natural components, emerged as the predominant printing technique in the bioprinting of liver models. Notably, the HepG2 hepatoma cell line was the most frequently employed liver cell type, despite acknowledged limitations. Furthermore, 51% of the printed models featured co-cultures with non-parenchymal cells to enhance their complexity. The included studies offered a variety of techniques for characterizing these liver models, with their primary application predominantly focused on toxicity testing. Among the frequently analyzed liver markers, albumin and urea stood out. Additionally, Cytochrome P450 (CYP) isoforms, primarily CYP3A and CYP1A, were assessed, and select studies employed nuclear receptor agonists to induce CYP activity.

**Conclusion:**

Our systematic scoping review offers an evidence-based overview and evaluation of the current state of research on bioprinted liver models, representing a promising and innovative technology for creating alternative organ models. We conducted a thorough examination of both the methodological and technical facets of model development and scrutinized the reporting quality within the realm of bioprinted liver models. This systematic scoping review can serve as a valuable template for systematically evaluating the progress of organ model development in various other domains. The transparently derived evidence presented here can provide essential support to the research community, facilitating the adaptation of technological advancements, the establishment of standards, and the enhancement of model robustness. This is particularly crucial as we work toward the long-term objective of establishing new approach methods as reliable alternatives to animal testing, with extensive and versatile applications.

## Highlights

• First evidence-based assessment of bioprinted liver models through a systematic scoping review.
• Extrusion-based printing is the predominantly used method in 3D bioprinting of liver models.
• Natural bioinks composed of protein and/or polysaccharide are most widely used in the field.
• HepG2 hepatoma cell line is most frequently employed liver cell type, despite known limitations.
• Identified reporting gaps enable improvements towards higher method reproducibility.

## Graphical abstract

**Figure:**
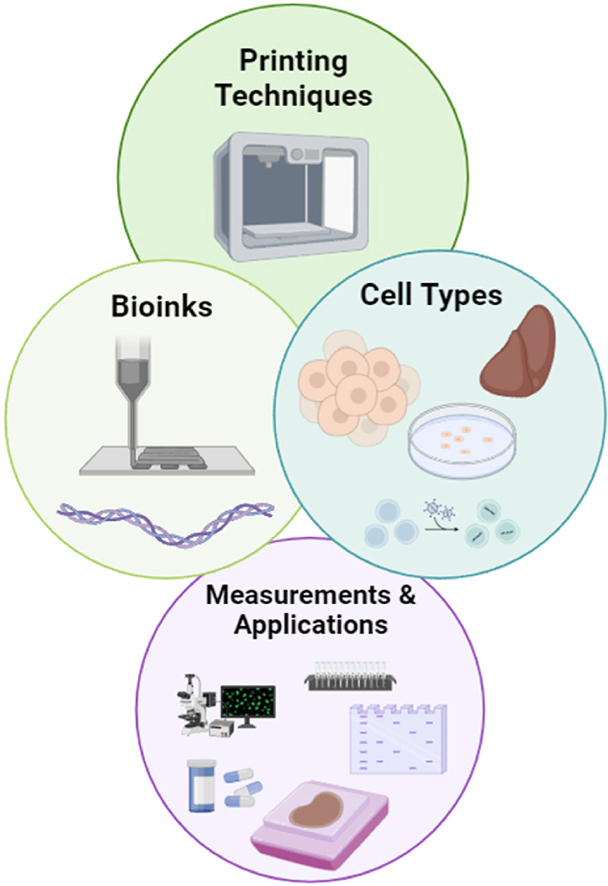

## 1 Introduction

The liver plays a central role in various physiological processes within the human body, e.g., detoxification, bile production, fat metabolism, storage of minerals and vitamins or monitoring innate and adaptive immunity [[1], [2], [3]]. Liver diseases are major causes of morbidity and mortality worldwide, leading to around 2 million deaths annually with an increasing tendency [4]. Over the past decades, various liver models have been developed, including 2D cell cultures, 3D organoids, microfluidic devices, and animal models to study chemical toxicity, liver function and liver diseases [[5], [6], [7]]. However, each of these models has its limitations in replicating the complex architecture and function of the liver.

**Fig. 1:**
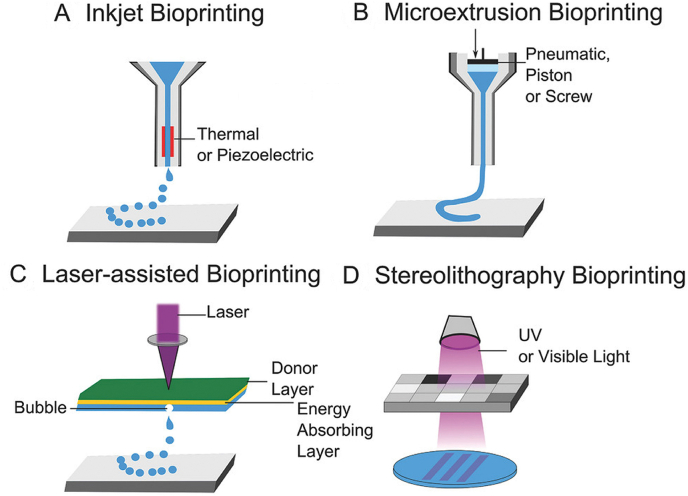
**Schematic representation of the common 3D bioprinting methods.** (A) Inkjet Bioprinting: Operates by ejecting bioink droplets from a print head onto a substrate in a controlled pattern, forming 3D structures through the layer-by-layer deposition of the bioink, (B) Microextrusion Bioprinting: Utilizes the extrusion of viscous bioink through a fine nozzle, depositing layers of material to build 3D structures, (C) Laser-Assisted Bioprinting: Involves using laser energy to generate microbubbles in a cell-containing bioink, propelling it onto a substrate with high precision, (D) Stereolithography Bioprinting: Employs a laser to selectively solidify layers of liquid photopolymer, typically hydrogel-based bioinks, in a controlled manner, layer by layer, to construct detailed 3D tissue structures. The figure is adapted from [12] used under a Creative Commons Attribution 4.0 International License: http://creativecommons.org/licenses/by/4.0/ [12].

In addition to these methodologies for the generation of artificial liver tissues, bioprinting has emerged as a particularly promising technology due to the ability to arrange cells with high spatial orientation and to combine different cell types [8,9]. The term ‘bioprinting’ comprises three-dimensional printing approaches that include living cells of human or animal origin during the printing process. In recent years, several bioprinting methodologies have been developed, such as extrusion-based bioprinting, inkjet-based bioprinting, laser-induced forward transfer, and photocuring-based bioprinting approaches [10,11]. Fig. 1 illustrates a schematic representation of the four common bioprinting methods, adapted from Foyt et al. [12]. The Inkjet-based bioprinting employs a non-contact method to deposit droplets of bioink onto a hydrogel substrate or culture dish. Inkjet-based bioprinting technique utilizes thermal or piezoelectric actuator methods for printing. Microextrusion bioprinting involves extruding biomaterials, typically in paste, solution, or dispersion form, using pneumatic pressure or plunger- and screw-based mechanisms through a microscale nozzle onto a stationary substrate. Laser-assisted bioprinting employs a pulsed laser to deposit biomaterials on a substrate, with the laser evaporating liquid biological materials from a ribbon onto a receiving substrate containing a biopolymer or cell culture medium. The stereolithography bioprinting is based on using a photo-sensitive bioink that solidifies upon illumination. The selection of a specific bioprinting technology for liver models depends on factors such as the desired applications, complexity of the models, and formulation of the printed materials. Further advantages, limitations and suitability of each bioprinting technology are covered in detail by Lima et al., 2022 [13].

Apart from the fabrication method, the properties and conditions of the bioink significantly influence the functionality and viability of the cells [14]. The bioink is composed of a hydrogel of single or blended components and must provide a tissue-specific microenvironment for the cells while ensuring suitable printability properties [15,16]. Generally, the printability properties can be adjusted by combining different materials, both natural (e.g., gelatin, alginate, collagen, or decellularized extracellular matrix dECM) and synthetic components (e.g., PEG or Pluronic), or by controlling the physicochemical conditions to enhance the biological performance of the bioinks [[17], [18], [19], [20]].

**Fig. 2:**
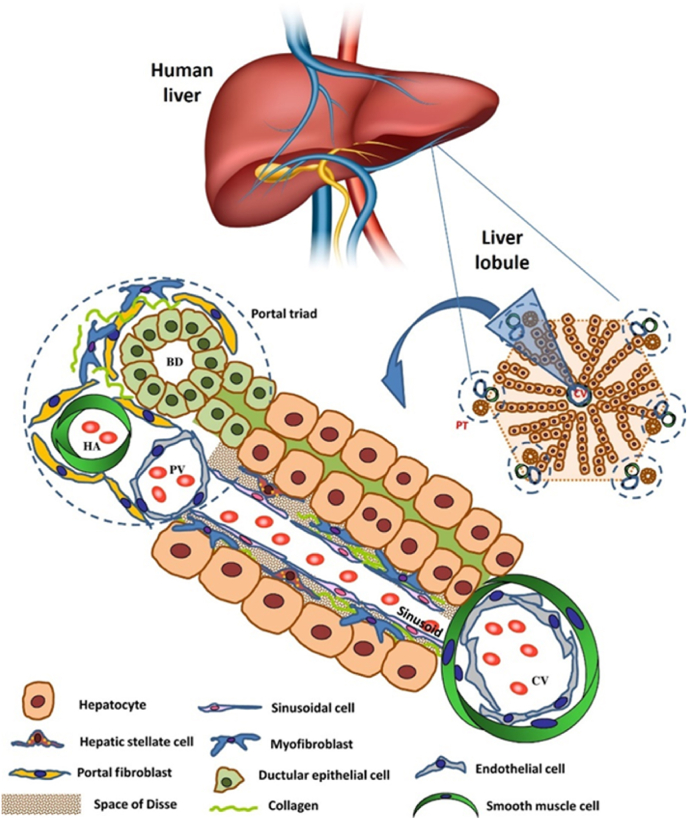
**Schematic representation showing the structure of liver lobule.** Each lobule has a hexagonal shape and is defined by six portal triads (PT) located at the corners and a central vein (CV) in the middle. The portal triad consists of a branch of the hepatic artery (HA), a branch of the hepatic portal vein (PV), and a bile duct (BD). The figure is modified from Cargnoni et al., 2018 [23] under a Creative Commons Attribution 4.0 International License: http://creativecommons.org/licenses/by/4.0/ [23].

**Fig. 3:**
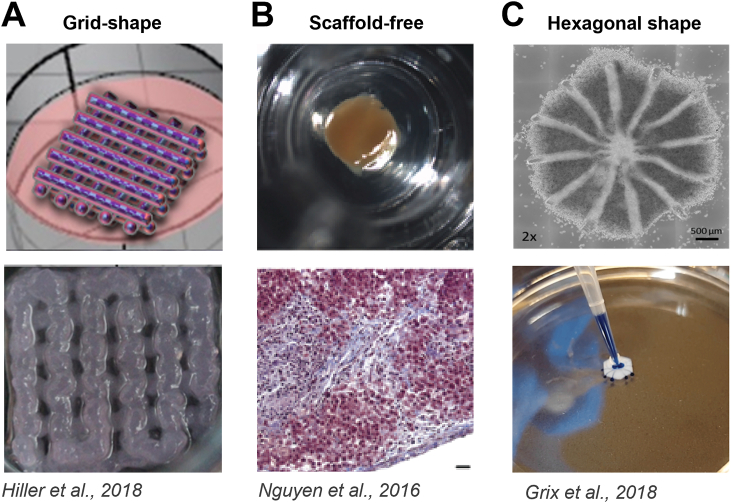
**Examples of reported liver models.** (A) Grid shape, (B) Scaffold-free assembly of cells, (C) Hexagonal shape. The figures are adapted from Hiller et al., 2018 [24], Nguyen et al., 2016 [25] and Grix et al., 2018 [26] under a Creative Commons Attribution 4.0 International License: http://creativecommons.org/licenses/by/4.0/.

The liver possesses a unique micro- and macromolecular structure, consisting of hepatic lobules with specialized cell types arranged in a specific order [21,22]. As shown in Fig. 2, each lobule is traversed by a central vein, and from this central vein, hepatocyte cords extend outward towards portal triads. Portal triads encompass three distinct structures: bile ducts, hepatic artery, and portal vein. Sinusoids, blood vessels lined by specialized fenestrated endothelial cells, separate hepatocyte cords. In the space of Disse, hepatic stellate cells are situated, while portal fibroblasts are located in the portal triad regions. Under conditions of injury, both hepatic stellate cells and portal fibroblasts can undergo activation, transforming into myofibroblasts, responsible for the production of extracellular matrix (ECM) [23]. ECM, synthesized by activated hepatic stellate cells and portal fibroblasts, provides structural support to the liver. In the context of 3D bioprinted liver models, incorporating ECM can mimic the native microenvironment, facilitating cell adhesion, and ensuring realistic tissue architecture. The complexity of the liver structure constitutes a challenge to produce a physiologically relevant bioprinted liver model. The shape of the printed constructs, the ability to perfuse the model, and the inclusion of different cell types contribute to the relevance and applicability of a printed model. Examples of reported 3D liver models are shown in Fig. 3.

Until today, the field lacks standardized guidelines and criteria for generating bioprinted liver models, resulting in variations in cell types, model complexity, and structural mimicry. The characterization of the physiological status of these models, through analysis of liver markers, and the activity and inducibility of drug-metabolizing cytochrome P450 enzymes, is essential for the recognition and broad application of alternative models.

The usage of animal components is still a limitation and challenge in the 3R research around human organ models. Many bioprinted tissues are cultured in medium containing fetal bovine serum (FBS) and contain animal-derived substances, such as Matrigel or collagen from porcine or rat sources. The pursuit of xeno-free organ models has been proposed to generate truly human organ models, avoiding the use of animal components. Advancing xeno-free organ models aligns with the goal of reducing animal usage in toxicity studies and could have substantial benefits for both scientific advancements and pharmaceutical drug development [[27], [28]].

To summarize the *status quo* on existing technological advancements in the field of bioprinted liver models and understand the variability in techniques used, we conducted a systematic scoping review, a method known for generating the highest quality of evidence based on a structured inclusion of publicly available scientific results, a reproducible methodology with minimized bias [29]. Here, we critically assessed all available knowledge on technical advances of 3D bioprinted liver models focusing on the following two research questions:

1 What are the different technological conditions and quality assurance measures for the 3D bioprinting of liver models?
2 What is the level of reporting in primary literature describing 3D bioprinting of liver models?

## 2 Methods

### 2.1 Protocol

This systematic scoping review was carried out in line with recommendations by the Joanna-Briggs-Institute and reported in line with PRISMA-ScR [30,31]. The protocol was prospectively registered on June 2^nd^, 2022, on Open Science Framework [32]. The only deviation from the planned methods was the addition of an exclusion criterion applied at the full text screening stage (”1. Articles describing the fabrication of scaffolds through printing that were subsequently seeded with cells, were excluded.“)

### 2.2 Information sources and search strategy

A comprehensive search was conducted on June 2^nd^, 2022, on MEDLINE Ovid, EMBASE Ovid and BioRxiv via API [33]. We did not use any additional filters or restrictions. Zotero (RRID:SCR_013784) was used to combine and deduplicate results from the databases and the included gray literature. We manually retrieved full text articles and combined main text and supplemental information.

### 2.3 Eligibility screening

Eligibility screening was conducted in two phases: Title and Abstract screening and full text screening using RAYYAN [34]. Two independent reviewers who were blinded to each other's decisions during the eligibility screen reviewed each study for inclusion/exclusion. Potential discrepancies in their decision were discussed at the end of the screening with the team and by consultation with a third reviewer.

### 2.4 Inclusion and exclusion criteria

The following inclusion criteria were applied:

1. **Article type and study design**: Articles had to include primary data that displayed the creation of a liver model with a printing technique involving liver cells. Articles were not subject to exclusion based on their study design. Moreover, articles that described liver model printing techniques from an engineering or medical perspective were included, e.g., case study, mechanistic study, etc.
2. **Liver cells**: Any liver cell types, whether human or animal, encompassing primary cells, stem cells or cell lines, including disease cell lines, were considered. Studies were incorporated even when multiple cell types were used, as long as liver cells were present in the final model. Primary studies that incorporated liver cells in the printing approach were included. Only liver models that included cells during the printing process were included.
3. **Printing**: All printing techniques were considered for inclusion if they described the utilization of liver cells. Such techniques encompassed, for instance, stereolithography, laser-assisted, inkjet-based, and extrusion-based methods. A bioprinted liver model was defined as a 3D cell culture containing liver cells, formed through a printing technique. Articles that did not describe an application of the model were considered eligible for inclusion in this systematic review. Irrespective of whether they described model viability tests, articles were included.

The following exclusion criteria were applied:

1. Articles describing the fabrication of scaffolds through printing that were subsequently seeded with cells, were excluded.
2. Primary studies that employed printing technology without liver cells being present in the final model were excluded.
3. Primary studies that described validation or set-up of printing techniques without describing an example printing of liver cells were excluded.
4. Articles that did not describe primary data displaying the creation of a liver model with a printing technique using liver cells were excluded. This exclusion encompassed types of articles such as reviews, systematic reviews, perspectives, opinion pieces or essays.
5. Articles written in a language other than English were excluded.

### 2.5 Data extraction

Before commencing the official data extraction, we conducted a pilot test of the data extraction form using MS Office Forms. Piloting involved 12 studies selected at random, which underwent review by two reviewers. Refinements of the data extraction form were implemented prior to transferring it to the SyRF platform (syrf.org.uk/; RRID:SCR_018907). All seven reviewers (A.A., D.W., J.B., B.T., D.T., A. Br., J.K.) have expertise in bioprinting and liver research, yet no one had previously conducted a systematic review. The reviewers were trained to do three trial extractions each. Two reviewers blinded to one another's decisions reviewed each study. The reconciliation was performed with the whole team and the methodological experts (A.B·B., M.H.) gave individual feedback. After the training phase, each reviewer independently extracted data from 23 studies (two randomly selected reviewers assigned to each paper). Discrepancies were reconciled by a third reviewer (A.A, D.W, M.H.) using a reconciliation add-on application to SyRF.

### 2.6 Data analysis

Data cleaning and visualization was performed in R using the following packages (Tidyverse (RRID:SCR_019186); Plotly (RRID:SCR_013991); see OSF for data and codebook [33].

### 2.7 Critical appraisal of reporting quality

Today, there is no agreed upon checklist assessing the reporting quality for studies describing *in vitro* model systems or bioprinting experiments. Here, we focus on essential details in the method sections, to allow a comprehensive understanding of the model at hand and the performed procedures. To assess the reporting quality of the presented *in vitro* studies we compiled a list of critical information that should be reported to comprehend the presented results and designed specific questions regarding reported details in the data extraction form (supplemental file 1 [33]).

## 3 Results

### 3.1 Selection of evidence

**Fig. 4:**
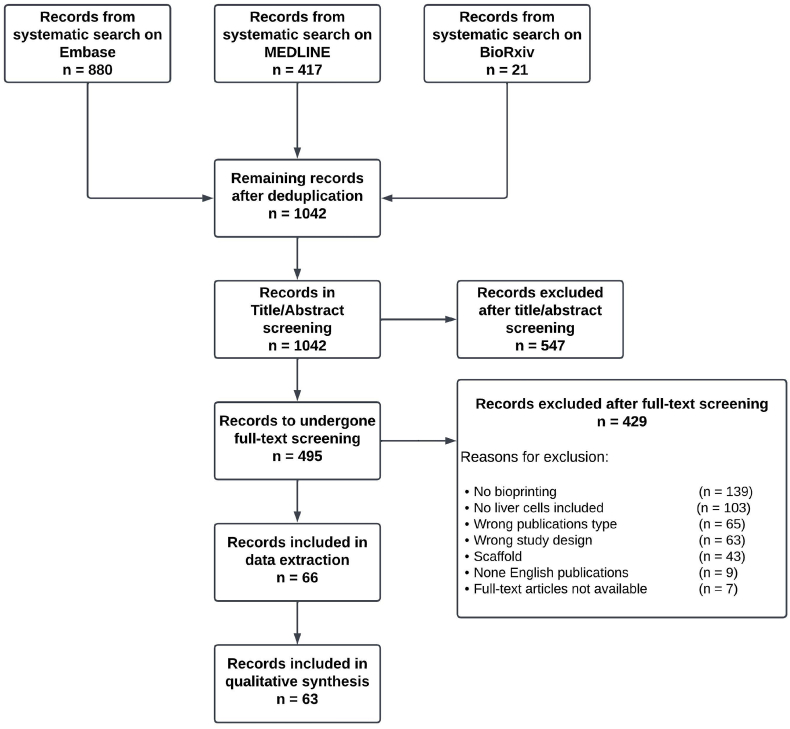
**Study selection flow chart**.

The searches on June 2^nd^, 2022, identified 1042 unique studies after deduplication (Embase, n = 880; Medline, n = 417; biorxiv, n = 21, example search strategy in supplemental file 2). The initial Title and Abstract screening identified 495 studies eligible for further review, with 547 studies ineligible. At full-text screening, 429 studies were found not eligible for the listed reasons (Supplemental table at OSF [33]). 66 studies were eligible for inclusion in the review and data were extracted. During data extraction, three additional studies were found not eligible during the data extraction process [[24], [25], [26],[35], [36], [37], [38], [39], [40], [41], [42], [43], [44], [45], [46], [47], [48], [49], [50], [51], [52], [53], [54], [55], [56], [57], [58], [59], [60], [61], [62], [63], [64], [65], [66], [67], [68], [69], [70], [71], [72], [73], [74], [75], [76], [77], [78], [79], [80], [81], [82], [83], [84], [85], [86], [87], [88], [89], [90], [91], [92], [93], [94], [95], [96], [97]]. This systematic review therefore includes information based on data extraction from 63 studies (Fig. 4). The reconciled outcome for each included study can be found on OSF [33].

The electronic database searches on Medline (880), Embase (417), and BioRxiv (21) resulted in 1318 articles. After deduplication, 1042 articles were processed for title and abstract screening (RAYYAN). 495 articles remained for full-text screening. Their eligibility had to be determined by screening the full-text articles. Here, 422 articles were excluded for the listed reasons. In total 66 studies were included for data extraction, of which three were found not eligible during data extraction. The extracted data from 63 articles contributed to the analysis.

**Fig. 5:**
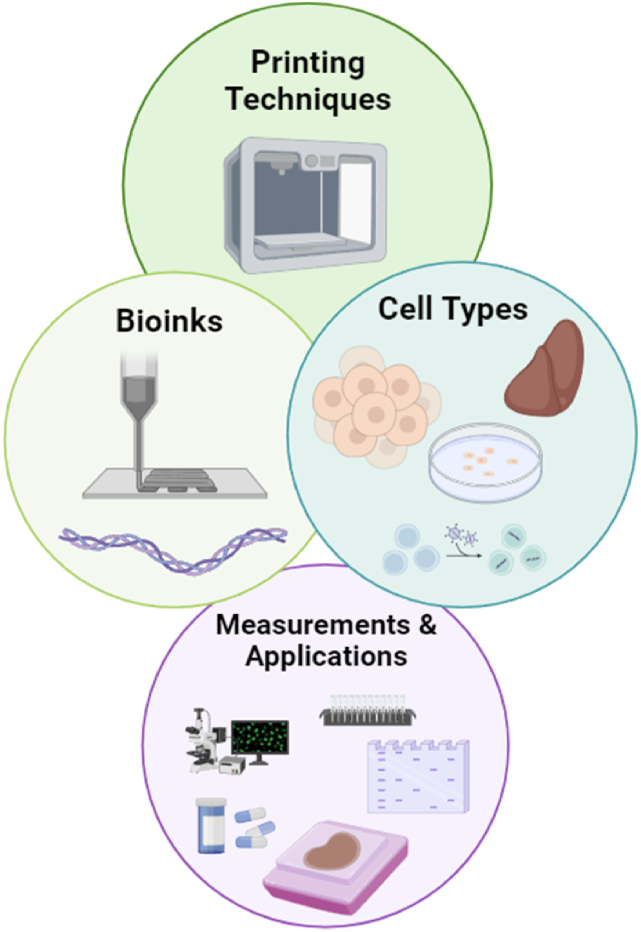
**Categories of data extraction.** We focused our data extraction on four main areas. The used printers and the printing technology, the applied bioink and its composition, the included cells, their source and culture conditions, and the conducted measurements and applications for the bioprinted liver model (Created with BioRender.com).

Here we present an evidence map on the practices in the field of 3D bioprinting of liver models. We are extracting evidence from purely *in vitro* studies that show primary results on liver model formation via bioprinting. As shown in Fig. 5, we extracted data on four main areas: the printing techniques, used bioinks, included cell types and the applications of the bioprinted liver models.

### 3.2 Printing techniques

**Fig. 6:**
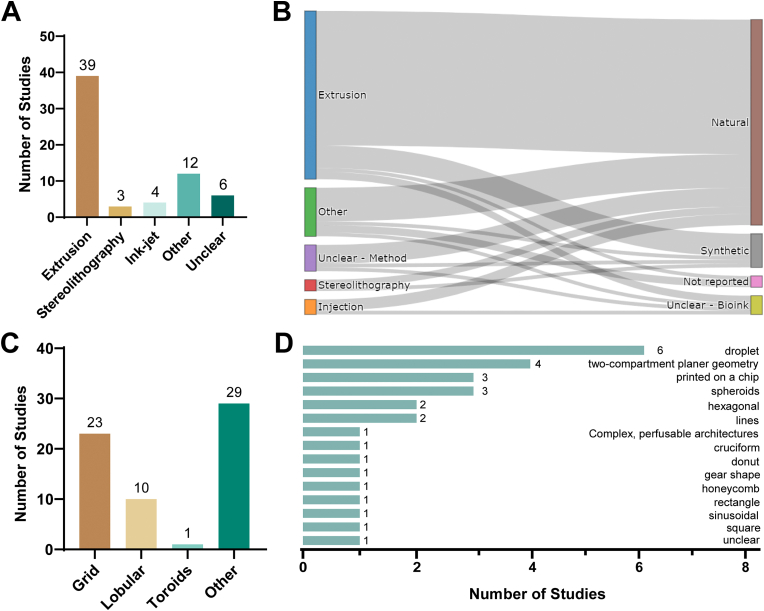
**Information on the applied printers and printed forms.** (A) Information on the kind of printing method. For 12 studies, reviewers chose ‘other’ printing techniques besides the ones we prelisted in the extraction form. For those, the reviewers manually extracted the reported printing techniques (supplemental file 2). (B) Information on the bioink composition (Sankey diagram, interactive version in online article or OSF [33]). Visualization of various printing techniques & ink combinations. On the left side the 72 models, printed with different techniques were plotted, and combined with the different bioink types (right side). Most studies of various printing techniques used natural components in the ink. (C) We predefined common forms that we expected to be printed. However, for 29 printing experiments the reviewers chose ‘other’ and extracted the information given by the authors of the study (D).

Commonly used bioprinting techniques are inkjet-based 3D printing, extrusion-based 3D printing and stereolithography [98]. The ink composition must precisely match the printing technique and printer at hand. Fig. 6 shows the extracted information for the reported printing techniques, printed shapes and applied inks in the investigated studies.

We screened the reported techniques employed for 3D bioprinting of liver models. Extrusion-based bioprinting emerged as the predominantly used method, appearing in 61% of the studies, while inkjet and stereolithography were employed in smaller proportions of around 6.3% and 9.5% of studies, respectively (Fig. 6A). The group ‘other’ summarized all printing techniques where reviewers were not able to categorize them into our prelisted techniques. The extracted ‘other techniques’ can be found in supplemental file 2.

**Fig. 7:**
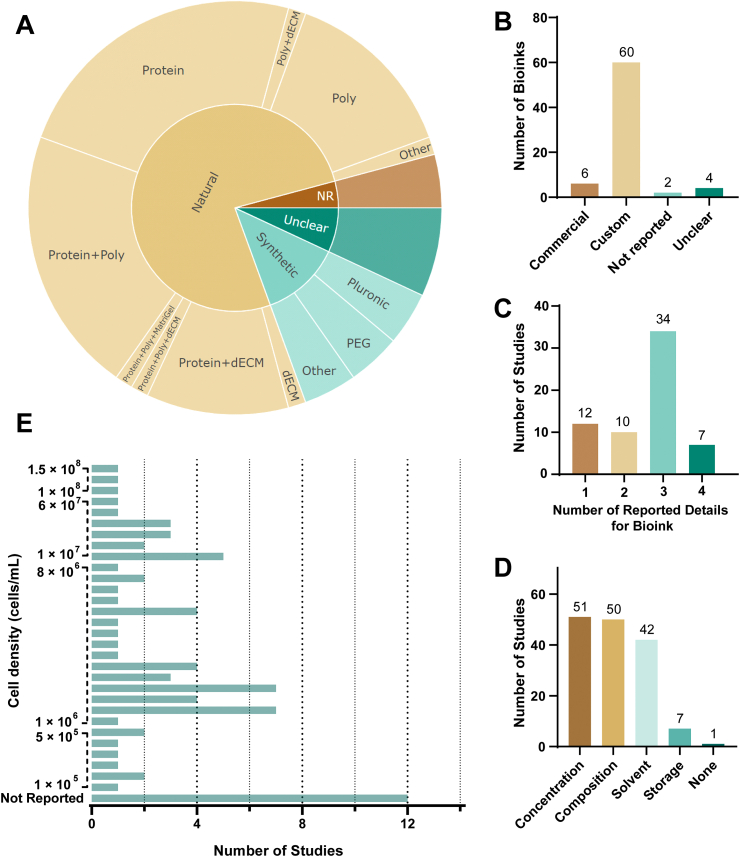
**Information on the bioinks**.

In Fig. 6B, we identified which bioink was employed for which kind of printing technique. The extracted data revealed that natural bioinks were predominantly used, regardless of the bioprinting technique employed. Most of the extrusion-based printing was performed with natural bioinks (80% of all studies), while in 13% of all extrusion-based printed models authors used synthetic bioinks, and for 7% it was unclear or not reported which type of bioink was used. For the stereolithography techniques, 60% of all printed models were created with natural bioink and 40% with synthetic bioink (additional details on the different components of natural and synthetic bioinks can be found in Fig. 7A).

Next, we assessed the printed shapes (Fig. 6C), as the shape has an impact on cellular nutrient uptake and biochemical signaling, thereby facilitating a better understanding of cell-cell interactions. Model complexity, however, varies with application requirements.

We predefined answer options for the most commonly printed shapes. The grid shape was reported as the predominantly printed structure among the analyzed studies, constituting 36% of the total, while in 16% of the studies, authors printed a lobular model. However, a significant portion of the reported forms could not be classified into these predefined shapes, and reviewers extracted individual shape details (Fig. 6D).

When looking for information on the printer, in 63% of the studies, authors provided information on the model and name. Moreover, in 63% of the studies they did not report the usage or name of a 3D modeling software.

### 3.3 Bioinks

Selecting the optimal bioink for 3D bioprinting is subject to many factors such as bioprinting method compatibility, suitability for the included cell type, printability with appropriate rheological behavior, and alignment with the targeted application [99,100].

From the 63 included studies, 72 different bioinks were reported. We differentiated between synthetic and natural bioinks and extracted information on their detailed ingredients. Out of the 72 reported bioinks, 55 inks were categorized as natural (Fig. 7A interactive and more detailed in online version on OSF [33]). Natural bioinks were further subcategorized as protein-based (31%), dECM-based (1.8%) or polysaccharide (Poly)-based (18%), or a combination of those (47 %). Most protein-based inks consisted of gelatin (70%). Most polysaccharide-based inks consisted of alginates (90%).

(A) Bioinks were categorized in natural (brown), synthetic (green), and their individual components and mixtures. We also categorized studies in unclear (dark green) or not reported (NR) (dark brown) – here the reviewers were not able to categorize the ink based on the information provided by the authors (Poly = polysaccharide, dECM = decellularized extracellular matrices, PEG = polyethylene glycol). A detailed and interactive sub-categorization can be found in the online version of this sunburst plot on OSF [33]. (B) Information regarding the source of the bioink were extracted: either commercially aquired or custom-made, and information on the number of details that authors provide were extracted (C), and their frequency (D). As an additional detail we extracted the reported cell densities of the bioinks. For 90% of the bioinks the cell density was reported and ranged between 1 × 10^5^ and 1.5 × 10^8^ cells/mL (E).

Synthetic bioinks only had a share of 12.5% of the reported 72 bioinks and the majority consisted of Pluronic (30%) or polyethylene glycol (PEG) (30%).

The great majority of all bioinks were custom formulated (83%), only 8% were commercially acquired, and for the remaining, we were not able to identify the source due to missing information (Fig. 7B).

To assess the level of reporting quality pertaining to bioinks, we attempted to extract the reported details on the bioinks in each study. Our analysis revealed that in most studies, authors reported three key pieces of information about the inks, which contained concentrations, compositions of the bioink and the solvents/media (Fig. 7C and D). We summarized the reported cell densities, which were provided for 90% of all bioinks (Fig. 7E).

### 3.4 Included cells and culture conditions

In 3D liver printing experiments, the choice of incorporated liver cells is essential, however also very challenging as the cells must emulate hepatocyte-like functionality and endure cell viability challenges during the printing process.

**Fig. 8:**
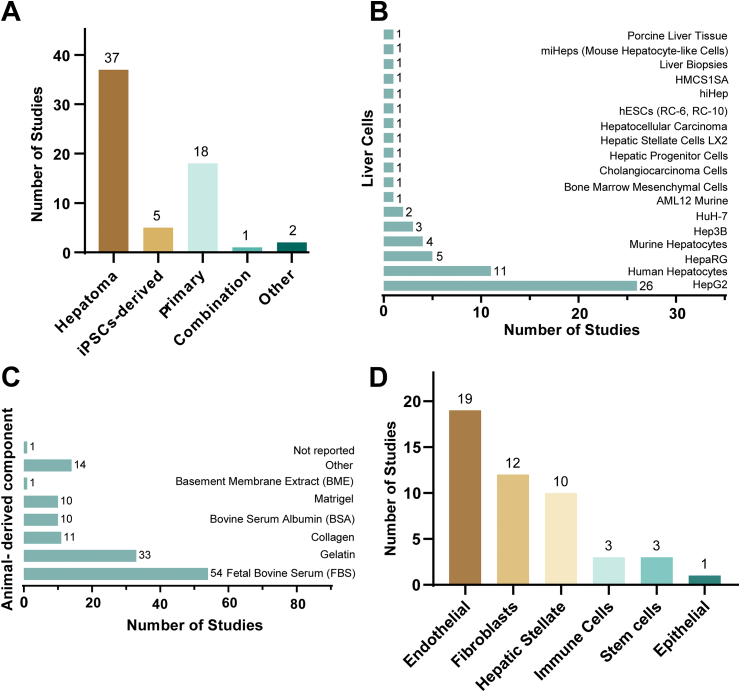
**Information on included cells and culture conditions**.

We therefore extracted the source of the cells from included studies. In 45 studies, authors used human cells, in four studies animal cells, and in 14 studies they reported models made from a combination of human and animal cells. Hepatoma cells were the most used liver cells in the printed models (47%) (Fig. 8A). Almost half of the reported models used HepG2 cells. Primary liver cells were the second most frequently reported cell type used. Fig. 8B shows a list of the applied liver cells and their frequency of use. Out of the 45 studies where models were based on human cells, none of the models were entirely xeno-free. In two studies it was not clearly reported if there were animal-derived components involved in the culture or model. All animal-derived additives are listed in Fig. 8C. Here, the category ‘other’ includes fibronectin, pepsin, dECM from bovine or porcine liver, goat and horse serum. In 32 out of 63 studies (51%) a co-culture with non-parenchymal cells was created (Fig. 8D), in 30 studies, authors worked with a monoculture and in one study, it was not clearly described how they cultured their liver cells. In four studies, authors included immune cells in their co-culture, all of which used primary macrophages. When we extracted information about additional information on the included liver cells, 89% of studies only provided one piece of information (e.g., name of cell line). In 51% of studies, authors reported the storage conditions for the models, whereas in all other studies it remained unclear how the models were stored.

We extracted information on the type of liver cells included in the liver model (A). In the majority of studies, authors used the hepatoma cell line HepG2 as main liver cells in their models (B). None of the investigated models were xeno-free and the included animal-derived components of the bioinks are listed in (C). The co-cultured liver models included the listed non-parenchymal cell types (D).

In terms of model complexity, the data showed that out of 63 studies, in 53 (84%) studies, authors did not report vascularization of their model, while in four studies they utilized perfusion, and in six studies they incorporated vascularization without perfusion. In seven studies, authors employed endothelial cells to build the vascular structure. In three studies vascularization without cells was reported. The data also revealed that in 59 studies (94%) oxygenation (hypoxia/normoxia) of the model was not addressed, while in three studies, authors did so in a descriptive manner and in one study by measurement.

### 3.5 Measurements and applications

**Fig. 9:**
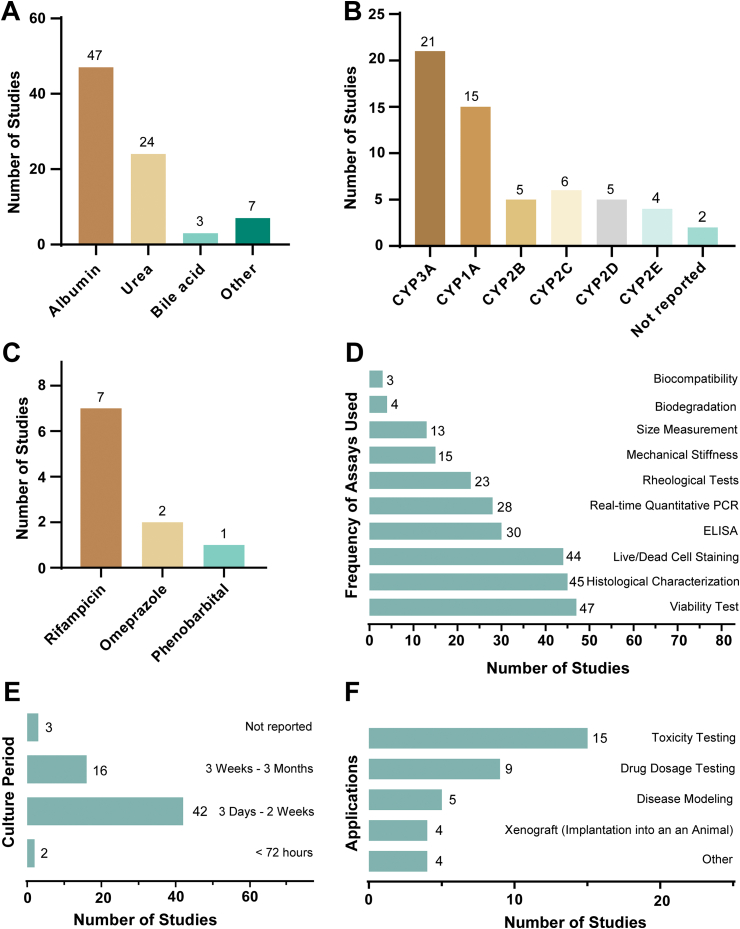
**Information on performed measurements in the model.** (A) Measured metabolites/liver markers in the bioprinted model. (B) Measured cytochrome P450 isoforms (CYP) in the bioprinted liver models. Often studies measured more than one CYP. (C) Applied CYP agonists. (D) Types of performed assays to assure the quality and integrity of the printed liver model. (E) Information on the reported duration of storage of the liver models. (F) Field of applications of the bioprinted liver models as reported in the respective studies..

To create a high-quality 3D bioprinted model, it is crucial to ensure the model's robustness and assess its mechanical and physiological properties. We therefore extracted information on the applied characterization techniques within the research studies. Various liver markers and metabolites were quantified. Albumin (75%) and/or urea (38%) production were the most frequently measured liver markers. Additionally, bile acids were measured as liver-specific metabolites as reported in three studies (Fig. 9A). In many instances, it was challenging to determine whether the samples were obtained from the supernatant, the culture media, or from the cell lysate, due to insufficient reporting.

In around half of the studies (30/63) authors measured cytochrome P450 (CYP) levels in the bioprinted liver models. The CYP family constitutes the most important group of hepatic drug-metabolizing enzymes and many *in vitro* liver models suffer from low expression of these enzymes. Fig. 9B lists the different CYPs and their frequency of measurement, with CYPs from the CYP3A and CYP1A families as the most frequently quantified CYP enzymes, being assessed in 21 and 15 studies, respectively. Physiologically, CYPs are regulated by several xeno-sensing nuclear receptors. To monitor functionality of xenobiotic induced CYP expression, in eight out of 63 studies they applied model receptor agonists to study the inducibility of CYPs (frequency shown in Fig. 9C).

We also extracted information on the different assays and characterization approaches that have been reported (Fig. 9D). In around two thirds of the studies, authors evaluated the physiology of the printed cells by cell viability or by conducting a histological analysis. The material properties of a bioink, its biocompatibility, its degradation profile, as well as rheological parameters like viscosity, and mechanical properties, have an impact on the overall features of a bioink formulation. In 23 studies, the rheological parameters of the bioinks were evaluated, while in only 15 studies, authors reported performed mechanical testing on their models. We investigated the storage conditions of the reported models. Here, we focused on the duration that authors reported the model to be in culture or under investigation in their experiments. We found that in 67% of studies, the authors stored the model between 3 days and 2 weeks, while in 25% of the studies, storage/culturing duration of the model was reported from 2 weeks to 3 months (Fig. 9E).

We summarized the broad fields of applications of the models (Fig. 9F). 37 studies presented applications of the models besides their formation. It appears that bioprinted liver models are primarily used in toxicity testings (41%) and drug dosage screenings (24%). In 14% of the application-focused studies, disease modeling was conducted, and the subsequent implantation of the model into an animal (Xenografting) was performed in 11%. The category ‘other’ summarizes cases were cell migration or viral infections were studied.

### 3.6 Reporting quality

We extracted information from included studies on the reporting quality in this field of research around alternative models and bioprinting. However, even for most of the other questions in our extraction protocol we included the answer option ‘not reported’ to indicate reporting gaps. As described in Fig. 6C we found that the printed forms are not standardized across the field, which presents a major challenge to model comparability. In 9% of the studies, authors did not report what type of bioink they were using in their printing process or where they acquired the ink (Figs. 6D and 7A&B). Additionally, we observed that it was often not possible to extract where the measured samples were taken from (e.g., cell extracts, media). 27 % of all studies do not provide any additional information on the included liver cells. While for common cell lines meta data, such as sex, age, health status of the donor is obsolete, authors are encouraged to include the RRID of cell lines, which has not been the case in any of the studies under investigation [101].

## 4 Discussion

In recent years, the 3D bioprinting technology around liver models has evolved rapidly. This progress has led to significant diversity in bioprinting techniques for liver models, bioink formulations, cell sources, and, consequently, substantial variation in techniques employed to evaluate the printed models. This wide range of different measurements and continuously evolving technologies have made it challenging to compare liver models across the literature. In this systematic scoping review, we aimed to thoroughly investigate the scope and nature of evidence on specific practices and reporting quality associated with bioprinted liver models in a structured manner.

Out of the 1042 studies initially identified, 63 were included in this review, according to predefined inclusion and exclusion criteria. Almost two-thirds of the screened liver models are generated by extrusion bioprinting, as one of the most popular printing techniques overall. A significant advantage of extrusion-based printing lies in its ability to fabricate designs with high cell densities, while offering flexibility in selecting from a diverse range of biomaterials for bioink composition.

One of the great strengths of bioprinting is the ability to generate complex structures with high resolution. Although widely used in the bioprinting field, it is surprising that a simple grid-like structure was the most used geometry for liver models, chosen by approximately one third of all studies analyzed. The natural hexagonal structure of liver lobes was reproduced in only 10 out of 63 studies. It should be noted that grid-like structures are often produced by the commonly applied extrusion technologies. Other printing technologies, such as stereolithography, provide higher resolution and can generate shapes that are more complex. Stereolithographic approaches, however, have substantial disadvantages such as the difficulties in multi-material printing and the need for a hydrogel that must be produced by optical cross-linking, commonly methacrylated gelatin [[102], [103], [104]]. It will thus be necessary to establish technologies that can produce the complex hexagonal structure of a liver lobe at high-resolution, without the restrictions imposed by stereolithography.

An optimal bioink formulation must meet specific biomaterial and biological criteria, including printability, mechanical properties, biocompatibility, and post-printing cell bioactivity. Therefore, natural polymers are commonly used as components of bioinks as they offer advantages over synthetics polymers, particularly in mimicking ECM composition [105]. We found that proteins and polysaccharides are widely used in 3D bioprinting of liver models, where combinations of Alginate, Collagen or Gelatin are the most popular bioink foundations.

Surprisingly, almost half of the studies used HepG2 cells for their liver models. Parenchymal cells are of utmost importance for the physiological function of the model. HepG2 cells, however, are widely considered not so suitable to model biological liver features, for example regarding drug and xenobiotic metabolism [106,107]. In contrast, HepaRG cells, which were used in only 5 out of 63 studies, resemble the expression patterns of important liver markers of natural hepatocytes more closely [108]. Still, these cells are hepatoma derived. Therefore, primary cells or hepatocytes differentiated from iPSCs might be a more suitable model for studying biotransformation of drug-metabolizing enzymes. However, the limited use of primary cells or differentiated stem cells is likely due to two difficulties: First, these cell types are more sensitive to cell damage during the printing process than established cell lines, and secondly, it is difficult to continuously obtain primary cells in sufficiently high numbers. Additionally, there is the variability between donors as another challenge using primary cells [109,110]. In addition, a physiologically relevant model should include non-parenchymal cells, such as liver sinusoidal endothelial cells, hepatic stellate cells, and Kupffer cells.

The scientific advances from 2D towards 3D cell culture models were a significant step. However, the 3D bioprinted monoculture models do not represent the signaling interactions between different cell types in actual liver tissue. In numerous research studies, authors have demonstrated that 3D bioprinted co-culture models outperform 3D bioprinted monoculture models in predicting drug-induced liver toxicity and drug metabolism in the liver [111]. The enhanced performance of 3D bioprinted co-culture models arise from their ability to recreate more realistic liver environments and characteristics, promoting interactions and signaling between different cell types. A key component to closely resemble the biological function of the liver is the introduction of multiple cell types (co-cultures) into the bioink. Our data showed that 51.5% of all published models incorporate more than one cell type.

Two main reasons are usually provided for the generation of bioprinted organ models: They are intended to replace animal models and thereby contribute to animal welfare, and they are human cell-based systems with potentially higher predictive value to human pathophysiology compared to certain animal models [27,112]. It was therefore a surprising finding of this systematic evidence map that in none of the studies the development of a xeno-free liver model was reported, i.e. in case the models were composed of human cells, they were still cultured with animal-derived components. Virtually all studies, which used human hepatocytes in their models cultured them in media containing fetal bovine serum (FBS). The extraction of serum from unborn calves of slaughtered cattle is, however, widely considered to be associated with suffering of the animals [113]. Furthermore, it places human cells in a complex mixture of growth factors and other components of animal origin, thereby producing a chimeric system, which will never represent human physiology. In addition to the culture medium, the bioinks used in most of the studies contained animal components such as collagen or gelatin. Again, this contradicts the attempts to produce models representing human physiology, and at the same time reduce animal suffering. We have therefore proposed the concept of clean bioprinting, which uses non-animal components only and therefore produces pure, xeno-free models [[27], [28]].

To fully harness the potential of bioprinting, it is essential to develop enhanced dynamic culturing approaches. Only 6% of the analyzed studies describe perfused bioprinted liver models. One essential part to resemble the biological function of the liver will require mimicking of the blood flow through the sinusoids, as well as retrograde bile flow through the bile canaliculi. Here again, high resolution of advanced bioprinting technologies can aid to produce physiologically relevant models.

Due to the central role of the liver during the metabolism and detoxification of endogenous and exogenous substances, the major applicability domain of bioprinted liver models lies in toxicity testing. Drug-induced hepatotoxicity is one of the major adverse effects during various drug treatments. In a milestone publication, Nguyen et al. demonstrated that a bioprinted liver model could detect hepatotoxicity of an antibiotic, which was found to be harmful to humans although it had passed all preclinical animal tests without the occurrence of adverse effects [25]. Animal models frequently do not produce reliable data for new drug candidates, and human models with high predictive power are therefore urgently required. Other applications of bioprinted liver models include disease modeling, e. g, liver cancer and liver fibrosis [67,72,79], or infection studies [24].

A wide range of assays and tests has been documented to assess the quality of the models discussed. Generally, the most reported assay involves testing cell viability, either through staining or measuring metabolic activity. The assessment of cell viability is crucial, serving as a key indicator for the successful printing process and the biocompatibility of the bioinks used. Furthermore, it is employed to evaluate the toxicity of administered drugs and toxins. Among the various liver biomarkers, albumin production is a pivotal marker in liver models, as evidenced by its frequent measurement in 75% of the studies. When assessing the toxicity of drugs and toxins, it is essential to examine drug-metabolizing enzymes. The cytochrome P450 enzymes (CYPs) stand out as the primary enzyme family capable of facilitating the oxidative biotransformation of most drugs. Among these enzymes, the CYP3A family has been the most extensively studied in the screened literature.

Throughout our analysis, we did not find any publications that deal with the use of AI in the assessment of 3D bioprinted tissue models. However, we assume that this technology will soon be implemented in bioprinting research efforts.

Alongside the scientific advances in the field of bioprinted liver models, we intended to track the quality of reporting in the investigated studies. While the reporting standards for animal experiments are well established [114], the methodological documentation for *in vitro* model systems strongly varies in quality. However, to be able to fully comprehend and reproduce the presented findings authors of primary experiments should provide sufficient information and share their detailed method descriptions [115]. We found that only two thirds of the studies sufficiently described the printing technique, the printer model and the software used. For one in ten included studies, we were not able to assess the components of the bioink, and for a third of the studies, the reported details on bioink composition/ratio/concentrations were incomplete. Especially since apparently the majority of bioinks are custom-formulated and not standardized, those details are important to transparently describe in scientific publications or open access protocols, to allow peers to build on the presented findings and adapt the technologies in other laboratories. Lack of reporting of key aspects of experimental design and conduct limit the ability for evidence syntheses such as systematic reviews to adequately investigate the variability and diversity in the field, and to understand where study design variables contribute to significant differences in the effectiveness of the 3D bioprinted liver models. Especially for research fields such as 3D liver bioprinting where the aim is the clinical application of the developed models, the sharing of detailed methodological procedures further enables trust and adaptation in the scientific community and regulatory agencies. This evidence map can be a foundation for the development of common reporting standards for bioprinted organ models in general.

The application of 3D bioprinting faces significant challenges when attempting to stimulate *in vivo* liver microenvironments. Firstly, the liver's complexity, characterized by multiple cell types and microstructures. Secondly, static models used in drug screening cannot accurately reflect the dynamic response of drugs under perfusion culture. The high throughput of 3D-bioprinted constructs poses limitations on their applications in this context. Thirdly, the current resolution of 3D bioprinting is inadequate for reproducing complex hepatic microenvironments. The scale of printed hydrogel structures is often too large to manipulate cells, and random cell distribution in the scaffold fails to ensure subtle anisotropy. Despite these challenges, 3D bioprinting remains a promising biofabrication strategy with the potential to revolutionize the medical field. Its innovation holds promises for creating artificial multi-cellular tissues and producing scaffolds for tissue and drug screening, organ transplantation, and regenerative medicine [116].

### 4.1 Limitations

We have conducted one of the first systematic reviews on a body of literature describing solely *in vitro* experiments, the first in the field of bioprinting. Most of our team are lab-based researchers with their primary expertise in bioprinting and liver toxicology, and limited experience in systematic reviews and meta research. The protocol development phase and the training phase for data extraction posed an intense phase with a steep learning curve guided by trained meta researchers. We are aware that our analysis is grounded on records that were sampled in June 2022, but we are convinced that the results presented here provide a sufficient overview of the state-of-the art in 3D bioprinting of liver models and can serve as a foundation for recommendations on future developments in this research field.

## 5 Conclusion

This comprehensive scoping review of bioprinted liver models' technological status can provide guidance for future advancements in printing techniques, model shapes, and cell selections. It serves as a blueprint for forthcoming studies evaluating new approach methodologies (NAMs), aiding the establishment of standards to expedite their validation for translational research and regulatory applications.

Advancing the development of meticulously designed and well-characterized bioprinted liver models is imperative for their broad application in drug screening and toxicity testing, thereby diminishing reliance on animal experimentation. The insights derived from this systematic scoping review reveal substantial diversity in cell sources, bioink compositions, and culture conditions within this emerging field of bioprinted liver models.

Notably, HepG2 cells, a hepatoma cell line subject to substantial criticism, emerge as the predominant source for liver cells in bioprinted liver models. Additionally, in only half of the models, hepatocytes were co-cultured with non-parenchymal cells, and only four models included a perfusion system, highlighting the ongoing deficiency in approximating physiological conditions within these systems. Strikingly, attempts to replicate the hexagonal shape of hepatic lobules in printed structures remain scarce, resulting in a wide array of diverse printed shapes that hinder model comparability. Notably, none of the studies reported a xeno-free liver model, signifying an area for further enhancement in these models, offering a robust alternative to animal experimentation.

## Funding

The presented work was supported by the Einstein Foundation and the Einstein Center 3R Berlin, (EC3R EZ-2020-597-2), the Chinese Scholarship Council (CSC, fellowship No. 201906780024 to D. W.), the “Stiftung zur Förderung der Erforschung von Ersatz-und Ergänzungsmethoden zur Einschränkung von Tierversuchen” (SET, P-075), and the Bundesinstitut für Risikobewertung (1328–568). ABB is supported by funding from the 10.13039/501100001663Volkswagen Foundation and Charité University Medicine - Charité 3R.

## Registration

The protocol was prospectively registered on June 2^nd^, 2022, on the Open Science Framework (OSF) Registry [[32], [33]].

## CRediT authorship contribution statement

**Ahmed S.M. Ali:** Conceptualization, Data curation, Investigation, Validation, Visualization, Writing – original draft, Writing – review & editing. **Dongwei Wu:** Conceptualization, Data curation, Investigation, Validation, Writing – review & editing. **Alexandra Bannach-Brown:** Conceptualization, Data curation, Formal analysis, Methodology, Project administration, Validation, Visualization, Writing – review & editing. **Diyal Dhamrait:** Data curation, Validation, Visualization. **Johanna Berg:** Conceptualization, Data curation, Writing – review & editing. **Beatrice Tolksdorf:** Conceptualization, Data curation, Writing – review & editing. **Dajana Lichtenstein:** Conceptualization, Data curation, Writing – review & editing. **Corinna Dressler:** Methodology, Writing – review & editing. **Albert Braeuning:** Conceptualization, Data curation, Investigation, Writing – review & editing. **Jens Kurreck:** Conceptualization, Data curation, Investigation, Writing – original draft, Writing – review & editing. **Maren Hülsemann:** Conceptualization, Data curation, Formal analysis, Investigation, Methodology, Project administration, Supervision, Validation, Visualization, Writing – original draft, Writing – review & editing.

## Declaration of competing interest

The authors declare that they have no known competing financial interests or personal relationships that could have appeared to influence the work reported in this paper.

## Data Availability

All relevant information, preregistration protocol [33], excluded studies with reasons, analysis code, executable figures and the raw data files can be found in the referenced OSF project.
