# Supplementary material for: 3D bioprinting of liver models: A systematic scoping review of methods, bioinks, and reporting quality
Source: Mater Today Bio. 2024 Feb 15;26:100991. doi: 10.1016/j.mtbio.2024.100991 (PMC10978534; doi:10.1016/j.mtbio.2024.100991)
Supplement: Multimedia component 1 [file mmc1.docx]

**Supplemental material:**

- **Supplemental_file_1**_data_extraction_form.docx [2]


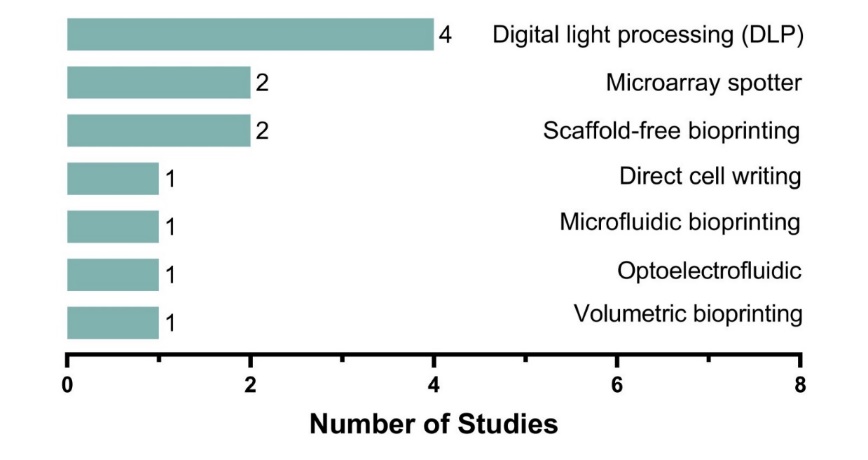


**Supplemental_file_2**: Additional printing techniques that were extracted by the reviewers additionally to the categories provided in Figure 6A.

1. exp Liver/
2. (hepat* or liver).hw,ti,ab,kf.
3. exp Printing, Three-Dimensional/
4. ((3D or "three dimensional") adj3 (print* or bioprint*)).ti,ab,kf.
5. (((3D adj2 model?) or (three dimensional adj2 model?)) and (print* or bioprint*)).ti,ab,kf.
6. (((3D adj2 cell?) or (three dimensional adj2 cell?)) and (print* or bioprint*)).ti,ab,kf.
7. 1 or 2
8. 3 or 4 or 5 or 6
9. 7 and 8

**Supplemental_file_3**: Example search strategy in Ovid MEDLINE(R)

- **Supplemental_table**_reonciled_data.xlsx [2]
- **Supplemental_table**_excluded_studies.xlsx [2]
