# Supplementary material for: 3D bioprinting of liver models: A systematic scoping review of methods, bioinks, and reporting quality
Source: Mater Today Bio. 2024 Feb 15;26:100991. doi: 10.1016/j.mtbio.2024.100991 (PMC10978534; doi:10.1016/j.mtbio.2024.100991)
Supplement: Multimedia component 2 [file mmc2.zip › Supplemental_file_1_data_extraction_form.docx]

Supplemental-file 3: formulated questions for data extraction and their corresponding answer options

In red the quality of reporting questions

1.1 Is the study included or excluded?

- Include
- Exclude

2.1 What is the origin of the cells in the liver model?

- Human
- Animal
- Both
- Not reported

2.1.1 If human, is the described liver model xeno-free/animal-free?

- Yes
- No
- Unclear

2.1.1.1 If not xeno-free, which additives are included in the study?

- Fetal Bovine Serum (FBS)
- Bovine Serum Albumin (BSA)
- Gelatin
- Collagen
- Basement Membrane Extracts (BME)
- Matrigel
- Cultrex
- Not reported
- Other

2.1.2 If animal, which of the following additives are included in the study?

- Fetal Bovine Serum (FBS)
- Bovine Serum Albumin (BSA)
- Gelatin
- Collagen
- Basement Membrane Extracts (BME)
- Matrigel
- Cultrex
- Not reported
- Other

2.1.3 If both, which of the following additives are included in the study?

- Fetal Bovine Serum (FBS)
- Bovine Serum Albumin (BSA)
- Gelatin
- Collagen
- Basement Membrane Extracts (BME)
- Matrigel
- Cultrex
- Not reported
- Other

2.2 What is the main type of liver cells included?

- Primary cells
- Induced pluripotent stem cells
- Hepatoma cells
- Other

2.2.1 How is the presented liver model cultured?

- Monoculture
- Co-culture
- Unclear

2.2.1.1 If co-cultured, what type of non-parenchymal cells are included?

- Immune cells
- Endothelial cells
- Hepatic stellate cells
- Other

2.2.1.1.1 Which kind of immune cells are present in the liver model?

- Macrophages
- T-cells
- B-cells
- Natural-killer cells
- Neutrophils
- Monocytes
- Other

2.2.1.1.2 What is the source of the immune cells in the liver model?

- Primary cells
- Cell lines
- Multiple sources
- Unclear

2.3 Select which information is provided about the liver cells?

- Name
- RRID
- Order number
- Manufacturer
- Isolation
- None

2.4 Which kind of meta data is available for the used liver cells?

- Sex
- Age
- Health status
- Common cell line
- None

3.1 Does the study present a vascularization of the model?

- Yes, with perfusion
- Yes, without perfusion
- No

3.1.1 If vascularization, which cells were used to build the vascular system? (with perfusion)

- Induced pluripotent stem cells
- Endothelial cells
- Stromal cells
- No cells
- Other

3.1.1 If vascularization, which cells were used to build the vascular system? (without perfusion)

- Induced pluripotent stem cells
- Endothelial cells
- Stromal cells
- No cells
- Other

3.2 Do the authors address hypoxia/normoxia/oxygenation of the liver model?

- Yes, descriptive
- Yes, by measurement
- No

4.1 What kind of printing method is used?

- Inject based
- Extrusion based
- Laser assisted
- Stereolithography
- Combination
- Unclear
- Other

4.1.1 Do the authors report the printer model name/number?

- Reported
- Not reported

4.1.2 What is the source of the printer?

- Commercial
- Modified commercial
- Self-made
- Unclear
- Not reported

4.1.3 What type of bioink was used?

- Natural
- Synthetic
- Unclear
- Not reported

4.1.3.1.1 If natural bioink: please choose the type.

- Protein based
- Polysaccharide based
- dECM based
- Unclear
- Other

4.1.3.1.2 If protein based: choose the type.

- Collagens
- Gelatin
- Fibrinogen
- Silk-fibrion
- Unclear
- Other

4.1.3.1.3 If polysaccharides based: choose the type.

- Alginates
- Gellan gum
- Hyaluronic acid
- Dextran
- Agarose
- Chitosan
- Unclear
- Other

4.1.3.1.4 If synthetic bioink: choose the type.

- Poly ethylene glycol (PEG)
- Pluronic
- Unclear
- Other

4.1.3.2 What is the origin of the bioink?

- Commercial (ready-to-use)
- Modified commercial
- Custom formulated
- Unclear
- Not reported

4.1.3.2.1 If commercial bioink, which information is provided for the bioink?

- Commercial name
- Manufacturer
- Batch number
- Storage conditions
- None

4.1.3.2.2 If modified commercial, which information is provided for the bioink?

- Commercial name
- Batch number
- Storage conditions
- None

4.1.3.2.3 If custom formulated, which information is provided for the bioink?

- Composition
- Concentration
- Solvent
- Storage conditions
- None

4.1.3.3 Which information is provided on the additives in the ink or culture?

- Concentration
- Manufacturer
- Order number
- None

4.1.3.4 Is the cell density of the bioink provided in the study?

- Yes
- No

4.1.3.4.1 Please specify the cell density (cells/ml)!

- (empty textbox to fill out)

4.1.3.4.1 Please specify the units of cell density (e.g., cells/ml; cells/µl; g/ml).

- (empty textbox to fill out)

4.1.4 What kind of forms are printed with this ink?

- Grid
- Lobular liver
- Cylinder
- Toroids
- Other
- None

4.1.5 Do the authors report the name of the 3D modelling software?

- Yes
- No
- Not applicable

5.1 Does the study report the storage conditions of the printed models?

- Yes
- No
- Unclear

5.2 How long were the liver models cultured after printing?

- < 72 hours
- 3 days - 2 weeks
- 2 weeks - 3 months
- 3 months - 1 year
- > 1 year
- Not applicable
- Not reported

5.3 Does the study describe quality-assuring assays for the printed model?

- Yes
- No
- Unclear

5.3.1 Which assays were performed to assure the quality of the liver model?

- Live/Dead Cell Staining
- Viability test
- Real-time quantitative PCR of liver markers
- Rheological test
- Biodegradation
- Mechanical stiffness
- Size measurement
- Histological characterization
- Biocompatibility
- Enzyme linked immunosorbent Assay (ELISA) of liver markers
- TEER (trans epithelial electrical resistance)

6.1 Which liver markers were analysed in the presented model?

- Alanine aminotransferase (ALT)
- Glutamic pyruvic transaminase (GPT)
- Aspartate aminotransferase (AST)
- Glutamic oxaloacetic transaminase (GOT)
- Alkaline phosphatase (ALP)
- Gamma-glutamyl transferase (GGT)
- Lactate dehydrogenase (LDH)
- None
- Other

6.1.1 Where were the liver markers measured?

- Media
- Cells
- Ink
- Unclear
- Not reported

6.2 Does the study analyse the Cytochrome P450 (CYP450) level in the print?

- Yes
- No

6.2.1 If yes, which cytochrome isoforms were analysed?

- Cytochrome P450 1A2 (CYP1A)
- Cytochrome P450 2A1 (CYP2A)
- Cytochrome P450 3A4 (CYP3A)
- Cytochrome P450 2B6 (CYP2B)
- Cytochrome P450 2C9 (CYP2C)
- Cytochrome P450 2D6 (CYP2D)
- Cytochrome P450 2E1 (CYP2E)
- Not reported
- Other

6.3 Have agonists of the receptors for the inducibility of CYPs been applied?

- Yes
- No

6.3.1 Please specify the agonists of the receptors that are applied.

- Pregnane X receptor (PXR)
- Constitutive androstane receptor (CAR)
- Aryl hydrocarbon receptor (AHR)
- Unclear
- Other

6.4 Which metabolites were analyzed in the study?

- Urea
- Bile acid
- Albumin
- None
- Other

7.1 Do the authors apply the model in the study?

- Yes
- No

7.1.1 Please select the field of application.

- Toxicity testing
- Drug dosage testing
- Implant / Medical surgery
- Xenograft (implantation into animal)
- Disease modeling
- Tissue engineering
- Other
