# Supplementary figures and images for: 3D bioprinting of liver models: A systematic scoping review of methods, bioinks, and reporting quality
Source: Mater Today Bio. 2024 Feb 15;26:100991. doi: 10.1016/j.mtbio.2024.100991 (PMC10978534; doi:10.1016/j.mtbio.2024.100991)

Supplemental-file 2: Additionally extracted printing techniques:


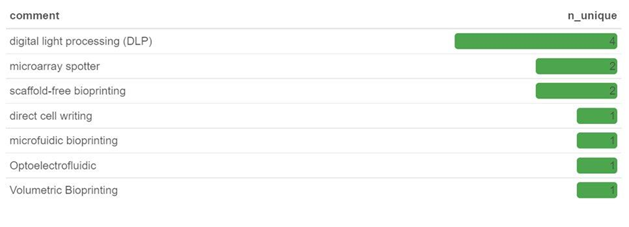


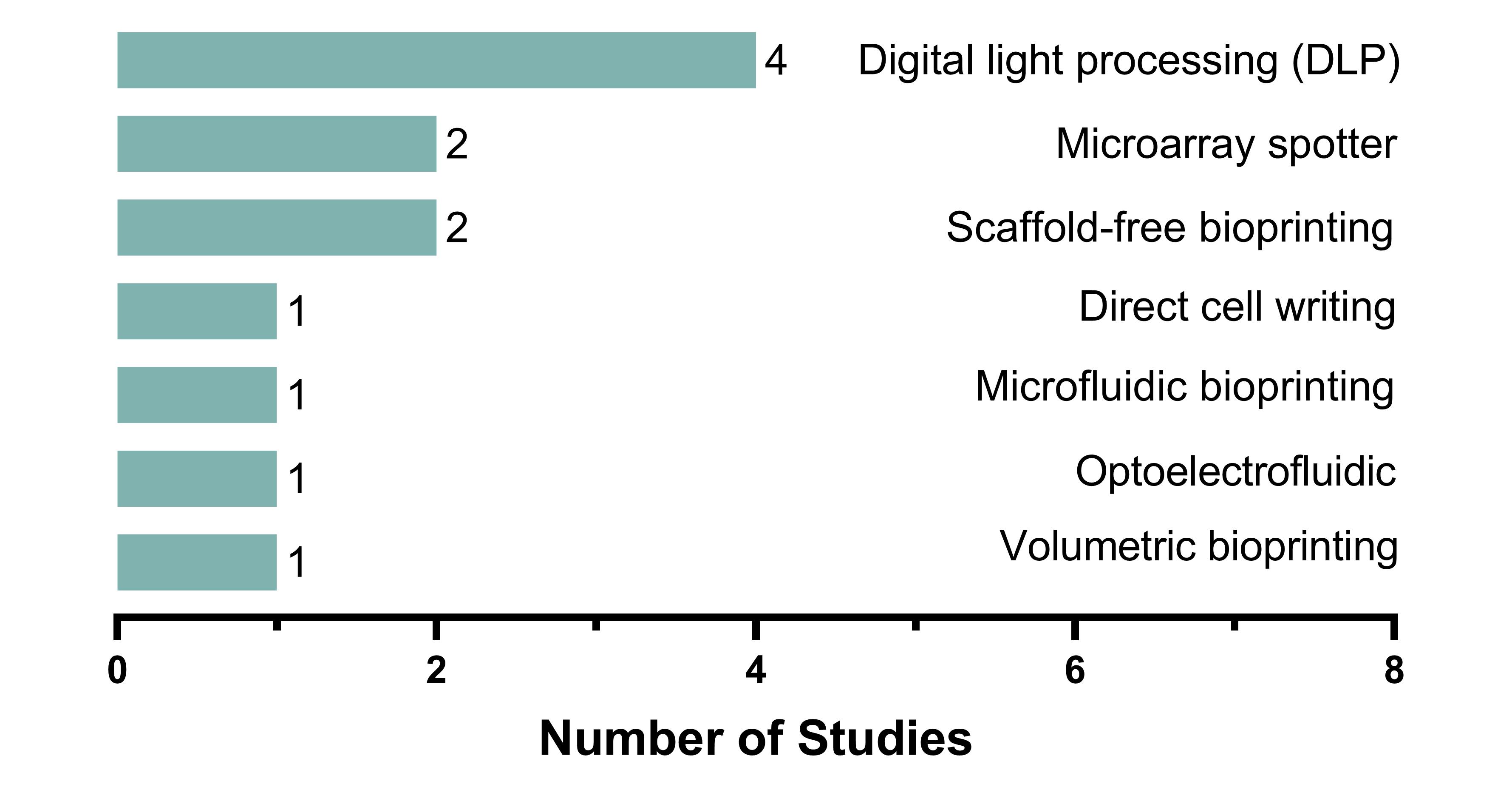

Supplement: Multimedia component 2 [file mmc2.zip › Supplemental_file_2_additional_printing_techniques.docx]

Supplemental-file 3: detailed Sunburst


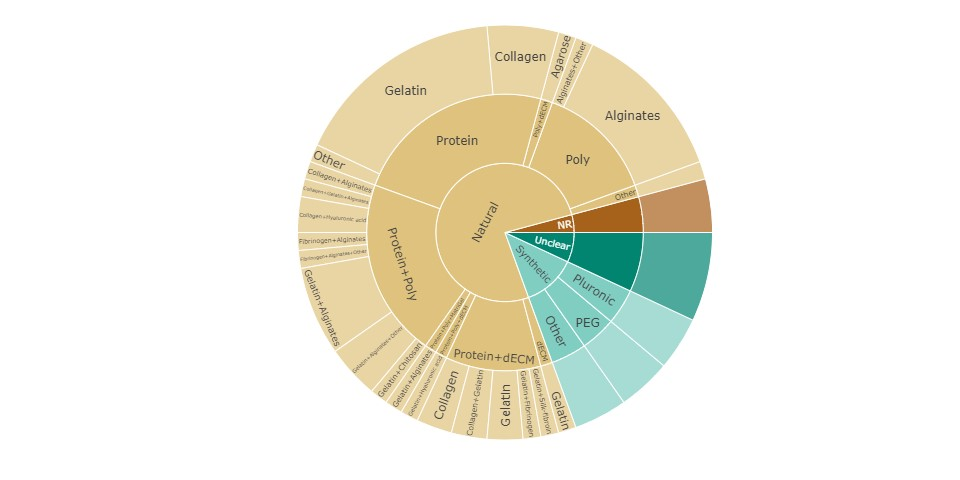

Supplement: Multimedia component 2 [file mmc2.zip › Supplemental_file_3_detailed_sunburst.docx]
